# Association of Apgar Score With Meconium Staining of Amniotic Fluid in Labor

**DOI:** 10.7759/cureus.12744

**Published:** 2021-01-16

**Authors:** Mehar Masood, Nadia Shahid, Zakia Bano, Fiza Ali Khan, Syeda Fariha Hussain, Hafiza Uroosa, Muzainah Khan, Adnan Anwar, Atif A Hashmi

**Affiliations:** 1 Obstetrics and Gynecology, Sindh Government Hospital, Karachi, PAK; 2 Obstetrics and Gynecology, Liaquat College of Medicine and Dentistry, Dar-Ul-Sehat Hospital, Karachi, PAK; 3 Obstetrics and Gynecology, Dow University of Health Sciences, Civil Hospital, Karachi, PAK; 4 Obstetrics and Gynecology, Jinnah Medical and Dental College, Karachi, PAK; 5 Medical Affairs, Aspin Pharma, Karachi, PAK; 6 Obstetrics and Gynecology, Karachi Medical and Dental College, Karachi, PAK; 7 Physiology, Al-Tibri Medical College, Isra University, Karachi, PAK; 8 Pathology, Liaquat National Hospital and Medical College, Karachi, PAK

**Keywords:** meconium staining, amniotic fluid, apgar score, parity

## Abstract

Objective

This study aimed to determine the association of Apgar score with meconium staining of amniotic fluid in labor.

Methodology

A retrospective observational study was carried out through the non-probability convenient sampling technique at the Department of Obstetrics and Gynecology for a duration of six months. Only those women were selected who had more than 24 weeks of gestation period. The women were excluded on the basis of risk factors for fetal distress and breech in late labor.

Results

A total of 216 pregnant women were selected from the labor room in this study. The mean age of the women was 26.57±4.28 years. The gestational age of the women was 36.09±4.11 weeks. Moreover, the mean parity of pregnant women was 1.68±2.53. It has been observed that the women who had meconium staining, the neonates of 144(77.4%) women showed the Apgar score of less than six at one minute. However, for the women without meconium staining, the neonates of only 15(50%) women showed the Apgar score of less than six at the one-minute interval with a significant association (p=0.02). With respect to age groups, a significant association of meconium staining with Apgar score was noted in the 21-30 years age group, whereas, no significant association was seen in other age groups. Similarly, a significant association of meconium staining and Apgar score was noted in primiparous women, whereas, no significant association was noted in multiparous women. No significant association of Apgar score and meconium staining was seen with respect to the mode of delivery.

Conclusion

The study has found a relation between the Apgar score and meconium staining of amniotic fluid and reported that the Apgar score of less than six at one minute was significantly associated with meconium staining of amniotic fluid.

## Introduction

The amniotic fluid is basically a yellow color fluid that is clear and found in the first 12 days after the female has conceived and is produced inside the amniotic sac. This fluid surrounds the baby, which is growing in the uterus and has major functions to perform, which leads to the healthy development of a fetus. The amount of amniotic fluid that is present inside the uterus is less but has a greater impact on the fetus. It acts as a protective system for both the mother and baby. Initially, the amniotic fluid consists of water which is obtained from the mother’s body afterward converted into the baby’s urine. Furthermore, there are some nutrients and important hormones along with anti-bodies that protect the fetus from bumps and injuries. Hence, if the level of amniotic fluid is monitored at a very low or high level it can be harmful to both mother and baby [1]. This part that is also known as the post-conception period has been critically advised to be taken extra care of both mother and child since the uterus is quite weak and the next immediate implantation may cause weak immunity, severe headaches followed by nausea, flatulence, cramps, and severe boating. Many women have experienced this condition in the initial trimester. One of the most pertaining factors that are usual during the implantation phase that may be experienced by the women is major mood swings and pressure on their lower abdomen. Meconium is a dark green substance that is composed of epithelial cells that are intestinal and contains mucus [2]. Meconium is produced in the fetal gut which is in small quantities as early as the tenth week of pregnancy approaches but, it is not excreted until about the 34th week of pregnancy [3]. It is also suggested that meconium is also secreted in response to fetal hypoxia or reduced clearance due to impaired swallowing or placental dysfunctions. Consequently, this had been used in labor to monitor the fluid which was separated from the womb. Meconium-stained amniotic fluid (MSAF) that occurs during delivery has long been known to be the indicator of adverse fetal and maternal effects such as meconium aspiration and perinatal asphyxia, contributing to perinatal and neonatal morbidity and mortality [4]. Meconium is a dense and black-green odorous substance first recognized during the gestation of the fetal intestine for approximately 12 weeks and deposited in the fetal colon [5].

The passage of meconium in newborns is an incident planned for development usually in the first 24 to 48 h after birth [6]. However, during pregnancy, the fetus can transfer meconium through the amniotic fluid for various reasons [7]. MSAF is rare before 37 weeks of gestation and increases with increasing gestational age [8]. The occurrence of MSAF during labor can cause adverse outcomes in the fetus, for instance, meconium aspiration syndrome (MAS) and perinatal asphyxia [9,10].

Meconium-stained liquor (MSL) is the passage of meconium in the antenatal or labor cycle by a fetus in utero [11]. In the intrapartum treatment guideline, meconium-stained amniotic fluid is graded as an important MSL and non-important MSL, according to the Royal College of Obstetricians and Gynecologists (RCOG) [12]. Non-important MSL is classified as a thin yellow or greenish tinged fluid; it contains non-particular meconium, while significant MSL is defined as a dense and stubborn, dark green or black amniotic fluid consisting of the meconium bumps [13].

Apgar score is a rapid method by which fetal well-being is evaluated against infant mortality. It is assessed based on five criteria (skin color, pulse rate, reflex, muscle tone, and respiratory effort) each with a scale of zero to two. Total score range from zero to 10. This study aimed to determine the association of Apgar score with meconium staining of amniotic fluid in labor.

## Materials and methods

A retrospective observational study was carried out through the non-probability convenient sampling technique at the Department of Obstetrics and Gynecology for a duration of six months. Women with labor pains that had three uterine contractions in 10 min, each lasting more than 30 seconds, with more than 24 weeks of the gestation period and established labor with spontaneous or artificial rupture of membranes were included in the study. However, the women were excluded on the basis of risk factors for fetal distress such as hypertension, diabetes mellitus, or previous cesarean section. Moreover, the women with heavy bleeding per vagina after 28 weeks of gestation (antepartum hemorrhage) along with the breach in late labor, i.e., baby presenting with buttocks or feet, and women with multiple pregnancies, i.e., more than one baby in utero were also excluded from the study. Women were categorized into age groups of <20 years, 21-30 years, and 30-40 years. The parity and mode of delivery were recorded.

The data were entered and analyzed on Statistical Package for Social Sciences (SPSS) Statistics version 26.0 (IBM Inc., Armonk, NY). Frequency and percentage were computed for categorical variables like the mode of delivery, parity, booking status, meconium-stained liquor and Apgar score less than six at one minute. Mean and standard deviation were computed for age, parity, and gestational age. For stratification, a cross-tabulation technique was used to control effect modifiers such as age groups, parity, and mode of delivery to observe the effect of an outcome. The chi-square test was used to find a proportional difference in Apgar score of less than 6 at one minute with meconium staining. P-values of ≤0.05 were considered statistically significant.

## Results

The mean age of the women was 26.57±4.28 years. The mean gestational age of the women was 36.09±4.11 weeks. Moreover, the mean parity of the pregnant women was 1.68±2.53 as shown in Table 1.

**Table 1:** Descriptive characteristics of women under study SD: standard deviation

Variable	Mean±SD	
		Age (Years)	26.57±4.28	
Parity	1.68±2.53	
Gestation Age (Weeks)	36.09±4.11	

It has been observed that the women who had meconium staining, the neonates of 144(77.4%) women showed the Apgar score of less than six at one minute. However, the women who did not have meconium staining, the neonates of only 15(50%) women showed Apgar score of less than six at the one-minute interval with a significant association (p=0.02) as shown in Table 2.

**Table 2:** Association of Apgar score of less than six at one minute with meconium staining

Meconium staining (n=216)	Apgar score <6 at one minute		P-value
	Yes	No	
Present	144(77.4%)	42(22.6%)	0.002
Absent	15(50%)	15(50%)	
Total	159	57	

With respect to age groups, the significant association of meconium staining with Apgar score was noted in the 21-30 years age group, whereas, no significant association was seen in other age groups. Similarly, the significant association of meconium staining and Apgar score was noted in primiparous women, whereas, no significant association was noted in multiparous women. No significant association of Apgar score and meconium staining was seen with respect to the mode of delivery (Table 3).

**Table 3:** Association of Apgar score and meconium staining with respect to different variables

Variables		Meconium staining	Apgar score <6 at one minute		P-value
			Yes	No	
Age groups (years)	<20	Present	20(87.0%)	3(13.0%)	0.16
		Absent	0(0.0%)	1(100.0%)	
	21 to 30	Present	114(77.6%)	33(22.4%)	0.011
		Absent	14(53.8%)	12(46.2%)	
	31 to 40	Present	10(62.5%)	6(37.5%)	0.54
		Absent	1(33.3%)	2(66.7%)	
Parity	Primipara	Present	67(81.7%)	15(18.3%)	0.004
		Absent	6(42.9%)	8(57.1%)	
	Multipara	Present	77(74.0%)	27(26.0%)	0.149
		Absent	9(56.2%)	7(43.8%)	
Mode of delivery	Emergency cesarean section	Present	115(82.1%)	25(17.9%)	0.22
		Absent	7(63.6%)	4(36.4%)	
	Spontaneous vaginal delivery	Present	29(63.0%)	17(37.0%)	0.12
		Absent	8(42.1%)	11(57.9%)	

## Discussion

In this study, we found a significant association of meconium staining of amniotic fluid with Apgar score, thus signifying the predictive value of meconium-stained amniotic fluid for fetal wellness. The increased incidence of meconium-stained amniotic fluid with advancing gestational age probably reflects the maturation of peristalsis in the fetal intestine. It has been observed that meconium-stained infants are usually born with clear amniotic fluid. A study by Duhan et al. for the period of three months to evaluate the presence of meconium at the onset of labor and its obstetric outcome, reported that out of the 1267 deliveries, 7.89% had meconium staining of liquor. In comparison with our study, the sample was much smaller with 216 cases and a high prevalence of meconium-stained amniotic fluid seen in 86.11% (186/216). The above study also reported adverse outcomes such as fetal heart rate abnormalities in women with meconium staining. Moreover, thick meconium staining was found in women with the higher cesarean section along with a low Apgar score at the one-minute interval. However, the frequency of Apgar score of less than six at one minute was reported to be elevated in meconium-stained and primiparous women. Moreover, both studies showed a strong relationship between meconium staining of amniotic fluid and Apgar score at the one-minute interval [14].

The age of women is also related to the development of MSAF. The study conducted in 2006 reported that women with more than 30 years of age were 5.6 times more susceptible to develop MSAF during labor than those less than 30 years. This is due to the fact that in older age, aging of uterine blood vessels and arterial stiffness results in insufficient placental perfusion leading toward the passage of meconium in the amniotic fluid [15]. On contrary, the other study found that women between 20 and 30 age groups had a high rate of Apgar score at one minute. Moreover, these women were also found with the presence of meconium-stained amniotic fluid [16].

Another study by Steer and colleagues revealed a relationship between meconium staining of the amniotic fluid and Apgar score. The sample size was larger (n=1219) compared to our study (n=216). The results revealed the presence of acidosis in the fetus whose mothers have MSAF during labor [17]. Alternatively, Grignaffini and colleagues found that in cases with MSAF, adverse fetal outcomes were absent with an Apgar score of less than seven at five minutes [18]. 

Another study reported that 48.8% of MSAF born babies had a low Apgar score of less than seven at one minute [19]. Similarly, the study by Bochner et al. reported that 40.3% of babies among the MSAF group had a low Apgar score at one minute compared to 3.9% in clear liquor babies [20]. Similarly, in our study, we found that the frequency of Apgar score of less than 6 at one minute was significantly higher in meconium-stained women.

Swain and Thapalial in 2008 predicted different associated syndromes in MSAF women. They further found significant risk factors associated with MAS, which include increased gestational age, increased cesarean section (LSCS), and low Apgar scores at 1 and 5 min [21]. On the contrary, a cross-sectional study conducted in 2004-2005 found no relation between MAS and type of delivery, and gestational age [22]. 

There were a few limitations to the study. First, a retrospective study design leads to the introduction of several confounding factors that cannot be controlled. Second, other sequel of meconium staining on fetal well being and other associated risk factors were not evaluated. Moreover, the sample size of the study was relatively small.

## Conclusions

We found a significant association of Apgar score at one minute with meconium staining of amniotic fluid. Moreover, after stratification of age, parity, and mode of delivery, we noted that the association of Apgar score and meconium staining of amniotic fluid was only significant in age groups between 21 and 30 years and primiparous women. More prospective studies are required to further explore the effects of meconium staining on neonates and the reasons for the low Apgar score.
